# Blocking the Farnesyl Pocket of PDEδ Reduces Rheb-Dependent mTORC1 Activation and Survival of *Tsc2-Null* Cells

**DOI:** 10.3389/fphar.2022.912688

**Published:** 2022-06-23

**Authors:** Marisol Estrella Armijo, Emilia Escalona, Daniela Peña, Alejandro Farias, Violeta Morin, Matthias Baumann, Bert Matthias Klebl, Roxana Pincheira, Ariel Fernando Castro

**Affiliations:** ^1^ Laboratorio de Transducción de Señales y Cáncer, Departamento de Bioquímica y Biología Molecular, Facultad de Ciencias Biológicas, Universidad de Concepción, Concepción, Chile; ^2^ Laboratorio de Investigación en Ciencias Biomédicas, Departamento de Ciencias Básicas y Morfología, Facultad de Medicina, Universidad Católica de la Santísima Concepción, Concepción, Chile; ^3^ Laboratorio de Proteasas y Cáncer, Departamento de Bioquímica y Biología Molecular, Facultad de Ciencias Biológicas, Universidad de Concepción, Concepción, Chile; ^4^ Lead Discovery Center, Dortmund, Germany

**Keywords:** Rheb, PDEδ inhibitor, Deltasonamide 1, *Tsc2-null* cells, mTORC1 signaling

## Abstract

Rheb is a small GTPase member of the Ras superfamily and an activator of mTORC1, a protein complex master regulator of cell metabolism, growth, and proliferation. Rheb/mTORC1 pathway is hyperactivated in proliferative diseases, such as Tuberous Sclerosis Complex syndrome and cancer. Therefore, targeting Rheb-dependent signaling is a rational strategy for developing new drug therapies. Rheb activates mTORC1 in the cytosolic surface of lysosomal membranes. Rheb’s farnesylation allows its anchorage on membranes, while its proper localization depends on the prenyl-binding chaperone PDEδ. Recently, the use of PDEδ inhibitors has been proposed as anticancer agents because they interrupted KRas signaling leading to antiproliferative effects in KRas-dependent pancreatic cancer cells. However, the effect of PDEδ inhibition on the Rheb/mTORC1 pathway has been poorly investigated. Here, we evaluated the impact of a new PDEδ inhibitor, called Deltasonamide 1, in *Tsc2*-*null* MEFs, a Rheb-dependent overactivated mTORC1 cell line. By using a yeast two-hybrid assay, we first validated that Deltasonamide 1 disrupts Rheb-PDEδ interaction. Accordingly, we found that Deltasonamide 1 reduces mTORC1 targets activation. In addition, our results showed that Deltasonamide 1 has antiproliferative and cytotoxic effects on *Tsc2-null* MEFs but has less effect on *Tsc2-wild type* MEFs viability. This work proposes the pharmacological PDEδ inhibition as a new approach to target the abnormal Rheb/mTORC1 activation in Tuberous Sclerosis Complex cells.

## 1 Introduction

The mechanistic Target of Rapamycin complex 1 (mTORC1) is a central regulator of cell metabolism, growth, and proliferation (Ben-Sahra and Manning, 2017). The mTOR protein is the catalytic subunit of the complex with serine/threonine kinase activity (Yang et al., 2013). In response to growth factors (Garami et al., 2003), nutrients (Long et al., 2005b; Miloslavski et al., 2014), and energy (Sancak et al., 2008; Hoxhaj et al., 2017), the small GTPase Rheb (Ras homolog enriched in brain) activates mTORC1, leading to phosphorylation of 4EBP1 (eukaryotic initiation factor 4E binding protein 1) and S6K1 (p70 ribosomal S6 kinase 1) (Castro et al., 2003; Avruch et al., 2009; Rapley et al., 2011). The 4EBP1 protein binds to eIF4E (eukaryotic initiation factor 4E) and inactivates this factor, whereas activation of mTORC1 inhibits 4EBP1, stimulating the cap-dependent protein synthesis by releasing eIF4E (Richter and Sonenberg, 2005). Moreover, S6K1 activation by mTORC1 leads to phosphorylation of ribosomal protein S6 and eEF2K (eukaryotic elongation factor 2 kinase), increasing ribosomal protein synthesis and cap-dependent protein translation (Holz et al., 2005). Therefore, mTORC1 activation positively controls protein synthesis.

Under several cell stressors or insufficiency of stimulus, mTORC1 signaling is turned off by a protein complex known as TSC (Tuberous Sclerosis Complex) (Huang and Manning, 2008; Dibble et al., 2012; Menon et al., 2014). TSC is a heterotrimeric complex formed by TSC1 and TBC1D7 proteins, with structural functions, and TSC2, a GTPase-activating protein (GAP) towards Rheb (Plank et al., 1998; van Slegtenhorst et al., 1998; Castro et al., 2003; Inoki et al., 2003; Zhang Y. et al., 2003; Dibble et al., 2012). Inactivating mutations in either the *TSC1* or *TSC2* gene are linked to TSC syndrome, an autosomal inherited disorder characterized by benign tumors known as hamartomas, most typically found in the brain, kidney, heart, lung, skin, and eye (Carsillo et al., 2000; Crino et al., 2006). The failure to inactivate Rheb, because of the loss of the *TSC1/2* genes, hyperactivates Rheb, resulting in constitutive mTORC1 activation and, consequently, aberrant cell growth and proliferation; therefore, underlying the pathogenic mechanisms of the TSC syndrome (Carsillo et al., 2000; Crino et al., 2006; Lacher et al., 2010). Thus, targeting mTORC1 signaling is a chosen strategy for treating this disease. The benefit of using the first-generation mTORC1 inhibitor (rapamycin) was initially demonstrated in a TSC animal model, with later clinical trials showing some improvement in TSC patients’ symptoms (Li et al., 2014). Everolimus, a rapamycin analog, is currently FDA-approved for some clinical manifestations of the TSC syndrome; however, resistance to this therapy prompt to search for better outcomes, exploring novel and more efficient strategies (Laplante and Sabatini, 2012; Li et al., 2014). Because of its relevance on the abnormal mTORC1 activation in TSC cells, targeting Rheb appears as a viable strategy to pharmacological inhibit mTORC1 signaling deregulated by TSC deficiency.

The small GTPase Rheb is a member of the Ras superfamily (Heard et al., 2014). Like other Ras proteins, Rheb presents a C-terminal CAAX sequence (C, Cysteine; A, Aliphatic Amino Acid; X, C-terminal Amino Acid) (Yamagata et al., 1994; Clark et al., 1997). The CAAX sequence directs a post-translational modification, resulting in farnesylation of Rheb (Yamagata et al., 1994; Clark et al., 1997). This farnesylation serves to anchor Rheb to endomembranes, such as the cytoplasmic surface of lysosomes and Golgi membranes (Takahashi et al., 2005; Buerger et al., 2006; Sancak et al., 2010; Hao et al., 2017). Farnesyltransferase inhibitors (FTIs) blocked the farnesylation of proteins like Rheb, negatively affecting their activities (Berndt et al., 2011; Spiegel et al., 2014). However, heterogeneous clinical results were obtained with FTIs, leading to a search for other strategies to inhibit small GTPase proteins (Berndt et al., 2011; Wang et al., 2017).

PDEδ (Phosphodiesterase 6δ) is a subunit of rod-specific cGMP phosphodiesterase, PDE6, but without catalytic activity (Gillespie et al., 1989; Zhang et al., 2004, 2005). In addition to its function in the PDE complex, PDEδ is a solubilization factor of farnesylated proteins, essential for the correct trafficking of its protein cargos (Ismail et al., 2011). In cells, inhibition of PDEδ caused mislocation of its farnesyl-cargos and impairment of the corresponding signaling (Siddiqui et al., 2020). PDEδ inhibitors are small hydrophobic molecules that associate with the PDEδ farnesyl-binding pocket, blocking the association with the cargo (Zimmermann et al., 2013). The location of Rheb on membranes and its intracellular distribution depends on the PDEδ (Schmick et al., 2015; Angarola and Ferguson, 2020). Thus, the PDEδ inhibition should affect Rheb cell distribution, leading to Rheb-mTORC1 signal transduction attenuation.

Although the first PDEδ inhibitor, Deltarasin, and the follow-up series of Deltazinones had antiproliferative effects and prevented association of PDEδ with KRas and Rheb, they were designed to target oncogenic Ras hyperactivation on Ras-driven cancer cells (Zimmermann et al., 2013; Papke et al., 2016; Klein et al., 2019). In this work, we used an overactivated Rheb/mTORC1 cell line (*Tsc2-null* MEF) to evaluate Deltasonamide 1, a new and improved PDEδ inhibitor (Martín-Gago et al., 2017). We showed that Deltasonamide 1 successfully disrupted Rheb-PDEδ interaction and downregulated mTORC1 signaling in *Tsc2-null* MEF cells. Furthermore, the Deltasonamide 1 treatment decreased cell proliferation and viability when Rheb/mTORC1 signaling was hyperactivated. Overall, pharmacological inhibition of PDEδ could negatively regulate mTORC1 signaling, being a potential new approach for downregulation of the Rheb/mTORC1 axis.

## 2 Materials and Methods

### 2.1 Reagents and Antibodies

Deltazinone 1 was purchased from MedKoo Biosciences (Morrisville, NC) and Deltasonamide 1 was purchased from Biozol Diagnostics (Eching, Germany). Rapamycin, protease inhibitor cocktail 1 (P8340) and phosphatase inhibitor cocktail 2 (P5726) were obtained from Sigma-Aldrich (St Louis, MO). The AccuRuler RGB plus protein ladder 26616) was purchased from MaestroGen Inc (Hsinchu City, Taiwan). Antibodies against 4E-BP1 (#9644), phospho-4E-BP1 (Thr37/46) (#9459), phospho-4E-BP1 (Thr70) (#9455), S6 ribosomal protein (#2317), phospho-S6 ribosomal protein (Ser235/236) (#2211), p70 S6 kinase (#9202) and phospho-p70 S6 kinase (Thr389) (#9206) were purchased from Cell Signaling Technology (Danvers, MA, USA). The primary antibody anti-β-Actin (sc-69870) was purchased from Santa Cruz Biotechnology (Dallas, TX, USA). Anti-mouse IgG-HRP conjugate (#1706516) and anti-rabbit IgG-HRP conjugate (#1706515) antibodies were purchased from Bio-Rad (Hercules, CA, USA). Anti-rabbit IgG Alexa488-conjugated (A-21467), anti-mouse IgG Alexa555-conjugated (A-21422) and Hoechst 33342 (H3570) were purchased from Invitrogen (Carlsbad, CA, USA).

### 2.2 Cell Culture

*p53−/− Tsc2+/+* mouse embryonic fibroblast (MEF) and *p53−/− Tsc2−/−* MEF cells (hereinafter referred to as *Tsc2-wt* and *Tsc2-null* MEFs) were kindly provided by David J. Kwiatkowski (Harvard Medical School, Boston, MA, USA). MEF cells were cultured in Dulbecco’s modified Eagles’s medium (DMEM) (Corning, New York, USA), supplemented with 100 μg/ml streptomycin (HyClone), 100 U/ml penicillin (HyClone), 10% fetal bovine serum (Biological Industries, Cromwell, USA) and incubated at 37°C in 5% CO_2_.

### 2.3 Western Blot

Proteins were extracted in cold lysis buffer (50 mM Tris-HCl pH 7.4; 10% glycerol; 200 mM NaCl_2_; 2.5 mM MgCl_2_; 1% Triton X-100; 1/100 protease inhibitors; 1/100 phosphatase inhibitors) and the protein concentration was determined by Bradford assay. Total proteins were denaturized in loading buffer (50 mM Tris-HCl pH 6.8; 2% SDS; 0.1% bromophenol blue; 10% glycerol; 1% β-mercaptoethanol) by boiling for 5 min, separated by SDS-PAGE, and transferred to Polyvinylidene fluoride (PVDF) membrane (MilliporeSigma, Burlington, USA). For immunoblotting, all primary antibodies were diluted 1:1.000, except for anti-β-Actin antibody (1:10000 dilution). Secondary antibodies were diluted 1:10000. Immunolabeled proteins were detected using ECL Western Blotting Detection Reagent (GE Healthcare, Chicago, USA) and visualized in Syngene PXi6 Documentation System (Frederick, MD, USA).

### 2.4 Immunofluorescence

Equal number of *Tsc2-null* MEF cells were seeded on 15 mm coverslips in 12-well plates and treated with 6 µM Deltasonamide 1 or DMSO (4 and 24 h). For GFP proteins, *Tsc2-wt* MEF cells were electroporated with 3 µg of pEGFP-C1-RhebWT (GFP-Rheb) or pEGFP-C1 (GFP only) using NEON™ Transfection System (Invitrogen). Briefly, 200.000 cells were electroporated with the 10 µl tips at 1100 V for 30 mseg (one pulse). Then, cells were seeded on 15 mm coverslips in 12-well plates (33.000 cells/well) and treated with 6 µM Deltasonamide 1 or DMSO for 24 h. Cells were fixed with 4% w/v paraformaldehyde (PFA) for 10 min and permeabilized with 0.1% Triton X-100 in Phosphate-Buffered Saline (PBS) for 30 min. Then, samples were blocked for 1 h with 3% BSA-PBS and incubated overnight with primary antibody (1:200 dilution) in a dark/wet chamber at 4°C. Next, fixed cells were incubated with secondary antibody (1:500 dilution) and Hoechst (1:250 dilution) for 1 h in the dark/wet chamber at room temperature. The coverslips were mounted on a slide with DAKO mounting medium (Agilent, Santa Clara, USA). Images were analyzed by confocal fluorescence microscopy using the LSM780 NLO Confocal Spectral Microscope (Carl Zeiss, Jena, Germany).

### 2.5 Yeast Two-Hybrid

**FIGURE 1:**
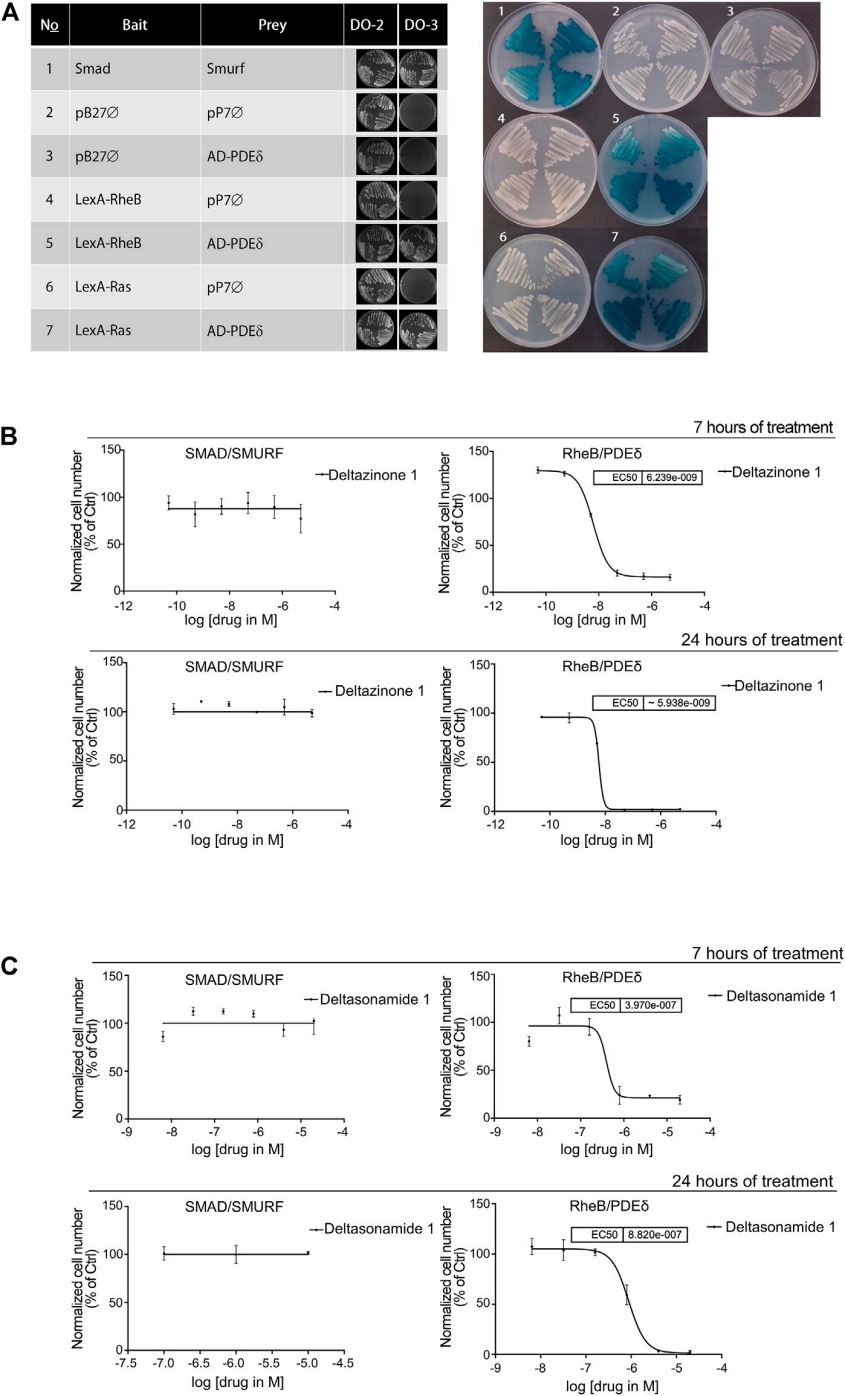
Deltazinone 1 and Deltasonamide 1 block the interaction between PDEδ and Rheb. **(A)**. *Left,* diploid yeast cells co-transformed with bait and prey constructs were grown on DO-2 or DO-3 medium. Only cells where bait and prey interact can grow on DO-3 medium. The interaction between Smurf and Smad was used as a positive control. *Right,* lacZ colorimetric assay. Diploid yeast cells co-transformed as in *left* were grown on DO-2 medium supplemented with X-gal substrate. As a result of bait and prey interaction, yeast cells express β-galactosidase and turn blue. The numerals refer to the numbering of bait and prey combinations shown in the left figure. **(B)**. Yeast two hybrid LacZ quantitative measurements for the interactions of Smad/Smurf and Rheb/PDEδ. Diploid yeast cells containing bait and prey constructs for Smad/Smurf or Rheb/PDEδ were grown on selective media lacking tryptophan and leucine in the presence of the β-galactosidase substrate ONPG and Deltazinone 1 at titrated compound concentrations (5 μM highest concentration) for 7 h (*upper panel*) or 24 h (*lower panel*) incubation prior to the measurement of β-galactosidase activity **(C)**. As in **(B)**. for Deltasonamide 1 (20 μM highest concentration). IC_50_, half-maximal effective concentration. Results are representative of at least three independent experiments. Data are expressed as the mean ± SD (*p* < 0.05).

The yeast two-hybrid screen was performed by Hybrigenics Services (Évry-Courcouronnes, France) with bait plasmids expressing Rheb, KRas, INPP5E, GRK1, Arl2 or Arl3 fusions to the LexA DNA-binding domain in pB27 vector and with pP7 prey vector expressing PDEδ fusions to the Gal4 Activation Domain. As a control, a yeast two-hybrid was performed using Smad (bait), Smurf (prey), and the empty vectors. Briefly, haploid yeast strains of opposite mating type were co-transformed with bait and prey constructs, as indicated in Figure 1. To verify positive clones, diploid cells were grown on selective media either lacking tryptophan and leucine (DO-2) or lacking tryptophan, leucine, and histidine (DO-3). To detect lacZ gene expression, diploid cells co-transformed with bait and prey constructs were grown on DO-2 medium supplemented with X-gal substrate. Co-transformed cells able to grow on DO-3). To detect lacZ gene expression, diploid cells co-transformed with bait and prey constructs were grown on DO-2 medium supplemented with X-gal substrate. Co-transformed cells able to grow on DO-2 medium and form blue colonies in the presence of X-gal were considered positive bait-prey interaction.

### 2.6 PDEδ Inhibitors IC_50_ Measurements

#### 2.6.1 Yeast Strain

Diploid yeast cells containing a centromeric expression of bait and prey constructs for Smad/Smurf, Rheb/PDEδ, and KRas/PDEδ, respectively, were grown on selective media DO-2 with β-galactosidase substrate ONPG (Ortho-Nitrophenyl-β-D-Galactopyranoside), and Deltazinone 1 or Deltasonamide 1 compound at titrated concentrations for 7 h or 24 h. The measure of β-galactosidase activity was evaluated by a colorimetric method. For INPP5E/PDEδ, GRK1/PDEδ, Arl2/PDEδ, and Arl3/PDEδ, yeast cells were treated with Deltasonamide 1 compound at titrated concentrations for 7 h.

#### 2.6.2 Mammalian Cell Line

Equal number of *Tsc2-null* MEF cells were seeded on 60 mm plates and treated with increasing concentration of Deltasonamide 1 for 4 h (Deltasonamide 1 highest concentration 8 µM). Levels of phospho-p70 S6 kinase (Thr389) (pS6K) were detected by Western blot and measured using densitometric analysis. Variations in β-galactosidase activity and levels of pS6K were used to calculate the half-maximal inhibitory concentration (IC_50_) of Deltazinone 1 and/or Deltasonamide 1.

### 2.7 Caco-2 Permeability Assay

To measure cellular permeability, Caco-2 cells were seeded on a transwell membrane in a 24-well format and grown for 3 weeks to form a monolayer of differentiated cells. Deltasonamide 1 or Deltazinone 1 were applied at a concentration of 10 µM in HBSS to either the apical (A) or basolateral (B) side of the Caco-2 cell monolayer and incubated for 2 h at 37°C. Compound concentrations on each side of the monolayer were determined by LC-MS/MS and the apparent permeability (P_app_) was calculated in the apical to basolateral (A→B) and basolateral to apical (B→A) directions according to the following equation:

$$P_{app\;\left(A\to B\right)}=\left(\text{Δ}C_{B}\times V_{B}\times 0.001\right)/\left(\text{Δt}\times \text{A}\times C_{t0,A}\right)$$

### 2.8 BrdU Assay

*Tsc2-null* MEF cells were seeded on 15 mm coverslips and treated with 20 nM rapamycin, 6 µM Deltasonamide 1, or vehicle (DMSO) for 24 h. BrdU (5-Bromo-2-deoxyuridine) labeling, and detection kit II (Roche, Basel, Switzerland) were used for the analysis. Briefly, cells were labeled 45 min with 50 µM BrdU and fixed for 20 min at 20 °C with a fixing solution (15 mM glycine pH 2.0, 70% ethanol). Then, cells were incubated with anti-BrdU antibody (1:20 dilution) and nucleases in incubation buffer (66 mM Tris, 0.66 mM MgCl_2_, 1 mM β-mercaptoethanol) for 30 min at 37°C in a wet chamber. For secondary antibody, Alexa 555 anti-mouse conjugated (1:500 dilution) was used. The nuclei were labeled with Hoechst 33342 diluted 1:250. Finally, the samples were mounted on a slide with DAKO mounting medium. At least 3 images were captured using Olympus CKX41 fluorescence microscope for each condition. BrdU positive cells were analyzed using ImageJ software.

### 2.9 XTT Assay

We used XTT based-Cell Proliferation Kit (Biological Industries, USA) according to the manufacturer’s instructions to assess cell viability and proliferation. Briefly, *Tsc2-null* or *Tsc2-wt* MEF cells were seeded in 96-well plates and grown in DMEM without phenol red (Biological Industries, Cromwell, USA) at 37°C in 5% CO_2_. Cells were incubated with 20 nM rapamycin, 6 µM Deltasonamide 1, or vehicle (DMSO) for 24, 36, 48, and 72 h, and XTT reagent was added at the end of each treatment. After 4 h of incubation, viable cells were detected by measuring the absorbance at 450nm/630 nm using TECAN Infinite^®^ 200 PRO microplate reader (Tecan Group Ltd. Männedorf, Switzerland). The results were expressed as a percentage of absorbance relative to the control group.

### 2.10 Live and Dead Cell Assay

We used Live/Dead™ Fixable Green Dead Cell Stain Kit (Invitrogen). Briefly, an equal number of *Tsc2-null* MEF cells were seeded in 12-well plates and treated with 20 nM rapamycin, 6 µM Deltasonamide 1, or vehicle (DMSO), and incubated at 37°C in 5% CO_2_ for 24 and 48 h. Cells were collected and incubated with LIVE/DEAD™ green dye (1:1000 dilution) and Hoechst 33342 (1:50 dilution) on ice for 30 min, washed twice with cold PBS, and suspended in 1% BSA-PBS. Finally, labeled cells were counted using the Countess™ II Automated Cell Counter (Invitrogen). Complementary, we also used CytoTox-Glo™ assay (Invitrogen, Carlsbad, CA) according to manufacture instructions. The results were expressed as the percentage of dead cells.

### 2.11 Statistical Analysis

All assays were done in triplicate and repeated three times. Data were analyzed by unpaired Student’s t-test or two-way analysis of variance (ANOVA) with Holm-Sidak correction to determine significant differences. Each experimental group was contrasted to its respective control group. A *p*-value less than 0.05 was considered statistically significant. Statistical analysis was performed with GraphPad Prism 6.

## 3 Results

### 3.1 PDEδ Inhibitors Block the Interaction Between PDEδ and Rheb

Deltasonamide 1 is a new small molecule developed to impair Ras signaling by disrupting its interaction with PDEδ (Martín-Gago et al., 2017). This compound showed more potent *in vitro* inhibition of KRas-PDEδ interaction than other PDEδ inhibitors, such as Deltazinone 1 (Papke et al., 2016). Deltasonamide 1 binds to PDEδ with a higher affinity and a K_D_ value of 203 pM vs. 8 nM for Deltazinone 1, as determined by competitive fluorescence polarization (Papke et al., 2016; Martín-Gago et al., 2017). To evaluate the effect of Deltasonamide 1 on Rheb-PDEδ association and compare it with that of Deltazinone 1, we performed a yeast two-hybrid assay using Rheb as bait and PDE as prey. In parallel, we analyzed the effect of the compounds on KRas-PDEδ association. The known interaction between Smurf and Smad served as a positive control of the assay (Zhang et al., 2001). All transformants grew in the absence of leucine and tryptophan amino acids. However, only transformants where the bait and prey proteins interacted could grow under additional selection pressure without histidine, indicating the association between Rheb or KRas and PDEδ (Figure 1A, *left*). Since the interaction between these proteins triggers the expression of β-galactosidase, we confirmed Rheb or KRas association with PDEδ by the appearance of blue colonies (Figure 1A, *right*). Treatment with Deltazinone 1 or Deltasonamide 1 for 7 and 24 h successfully blocked the interaction between Rheb and PDEδ, but the interaction between Smad and Smurf was not affected (Figure 1B-C). As expected, both compounds also blocked the interaction between KRas and PDEδ (Supplementary Figure 1). Despite the higher affinity of Deltasonamide 1 for PDEδ, Deltazinone 1 showed a lower IC_50_ for the Rheb-PDEδ interaction than Deltasonamide 1 under acute treatment (7 h, IC_50_ = 0.006 vs 0.397 µM) which could reflect different capabilities of the compounds to permeate through the yeast cell wall. To verify the permeability issue, we conducted *in vitro* permeability measurements using Caco-2 cells. Indeed, Deltazinone 1 demonstrated a much better permeation of differentiated Caco-2 cells as judged from the measurement of the apparent permeability (P_app_) from the apical to the basolateral side (P_app__(A→B)_ = 41.1 × 10^−6^ cm/s) compared to Deltasonamide 1 (P_app__(A→B)_ = 1.6 × 10^−6^ cm/s) while the efflux of Deltazinone 1 from differentiated Caco-2 cells as calculated from the ratio of P_app_ values from the basolateral to the apical side versus the apical to the basolateral side ( $P_{app\;\left(B\to A\right)}/P_{app\;\left(A\to B\right)}$ ) was much lower than the efflux value calculated for Deltasonamide 1 (Deltazinone 1 ratio, $P_{app\;\left(B\to A\right)}/P_{app\;\left(A\to B\right)}$ = 0.6; Deltasonamide 1 ratio, $P_{app\;\left(B\to A\right)}/P_{app\;\left(A\to B\right)}$ = 8.8; see Table 1) suggesting that Deltasonamide 1 is a substrate for efflux ABC transporters in Caco-2 cells which are also active in yeast cells (Piecuch and Obłak, 2014). The IC_50_ for Deltazinone 1 did not change after 24 h of incubation (24 h, IC_50_ = 0.006 µM), while the IC_50_ for Deltasonamide 1 was slightly higher (24 h, IC_50_ = 0.882 µM) (Figure 1B-C), which we do not consider to be a significant change in activity. Additionally, we performed yeast two-hybrid assays to test the effect of Deltasonamide 1 on the association of PDEδ with other known cargo proteins, like INPP5E and GRK1 (Zhang et al., 2004; Baehr, 2014). Deltasonamide 1 required a high concentration of 10 µM to affect the interaction of PDEδ with these proteins (Supplementary Figure 2A, B). Compared to the IC_50_ of 0.397 µM for Rheb and 0.923 µM for KRas after 7h, these results indicate that Deltasonamide 1 preferentially prevents complex formation between Rheb or KRas with PDEδ. Mechanistically, this selectivity can be explained by the higher affinity of both farnesylated-INPP5E and -GRK1 for the prenyl binding pocket of PDEδ, determined by two C-terminal amino acid residues at the -1 and -3 positions relative to the farnesylated cysteine (Fansa et al., 2016; Fansa and Wittinghofer, 2016). Thus, whereas INPP5E and GRK1 have affinities in the low nanomolar range, the K_D_ values of KRas and Rheb are submicromolar (Fansa et al., 2016; Fansa and Wittinghofer, 2016). Applying the yeast two-hybrid system, we also measured the ability of Deltasonamide 1 to disturb binding of PDEδ to the small GTPases Arl2 or Arl3 (Arf-like GTPase), both known to function as release factors for PDEδ cargos (Hanzal-Bayer et al., 2005; Ismail et al., 2011). In the GTP-bound state, Arl2 and Arl3 bind PDEδ via a hydrophobic pocket, inducing a conformational change in PDEδ that leads to the release of PDEδ-bound cargo and its correct intracellular re-location (Hanzal-Bayer et al., 2002; Ismail et al., 2011; Schmick et al., 2015). Again, we observed interference of Arl2/PDEδ or Arl3/PDEδ complex formation only at a high Deltasonamide 1 concentration of 10 µM (Supplementary Figure 2C, D). In yeast, we conclude that Deltasonamide 1 interferes with Rheb/PDEδ or KRas/PDEδ complex formation at sub-micromolar concentrations that have little or no impact on PDEδ binding to high-affinity cargo like INPP5E or GRK1 or the cargo release factors Arl2 and Arl3.

**TABLE 1:** Caco-2 cellular permeability values for Deltasonamide 1 and Deltazinone 1.

	Deltasonamide 1	Deltazinone 1
Caco-2 _(pH 6.5/7.4)_ P_app__(A→B)_ × (10^−6^ cm/s)	1.56	41.1
Caco-2 _(pH 6.5/7.4)_ P_app__(B→A)_× (10^−6^ cm/s)	13.67	25.1
Caco-2 _(pH 6.5/7.4)_ Ratio (P_app__(A→B)_/P_app__(B→A)_)	8.8	0.6

### 3.2 Deltasonamide 1 Downregulates the mTORC1 Signaling Pathway

**FIGURE 2:**
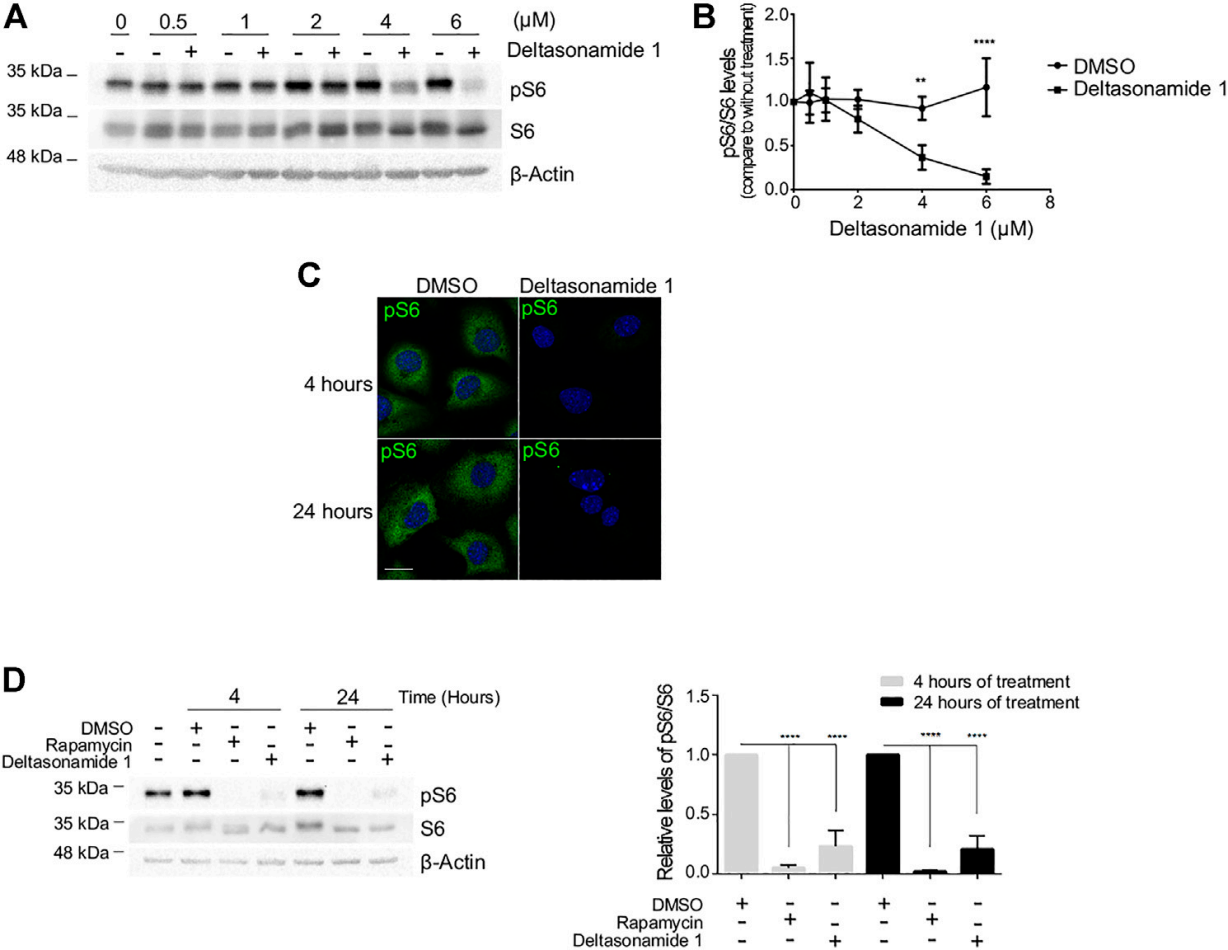
Deltasonamide 1 decreases S6 phosphorylation levels in *Tsc2-null* MEFs **(A)**. *Tsc2-null* MEFs treated with DMSO or Deltasonamide 1 (0.5–6 µM) for 24 h. Lysates were characterized by immunoblotting of the indicated proteins. pS6, S6 total and β-actin (loading control) **(B)**. Densitometric analysis of pS6 levels normalized to total S6 levels **(C)**. *Tsc2-null* MEFs treated with DMSO or Deltasonamide 1 (6 µM) for 4 and 24 h pS6 (green) levels were analyzed by immunofluorescence and confocal microscopy. Hoechst stained the nuclei (blue) **(D)**. Left, *Tsc2-null* MEFs treated with DMSO, rapamycin (20 nM), or Deltasonamide 1 (6 µM) for 4 and 24 h. Lysates were characterized by immunoblotting of the indicated proteins. Right, densitometric analysis of pS6 levels normalized to total S6 levels. Results are representative of at least three independent experiments. Data are expressed as the mean ± SD (*p* < 0.05).

**FIGURE 3:**
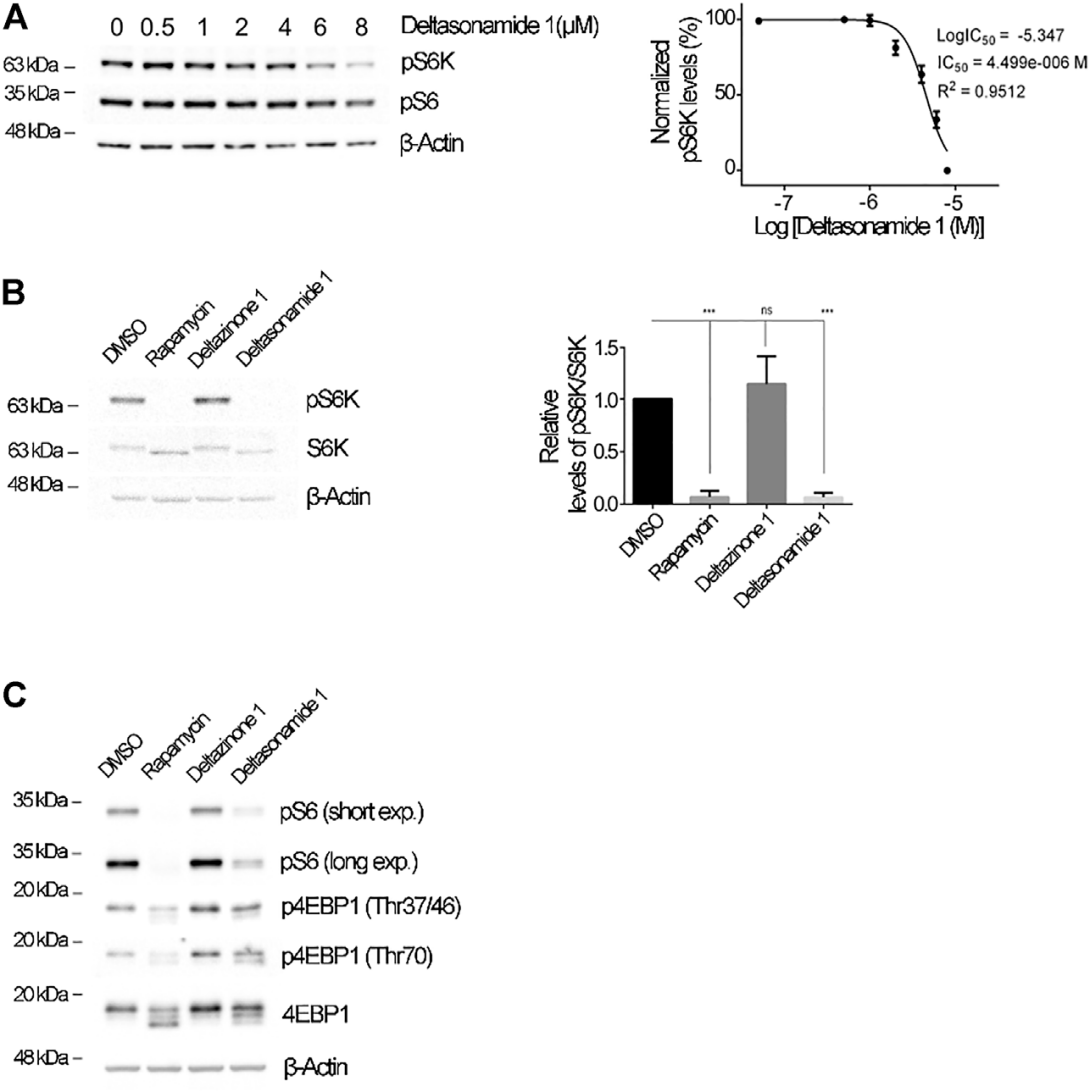
Deltasonamide 1 inhibits the mTORC1 signaling pathway in *Tsc2-null* MEFs **(A)**. *Tsc2-null* MEFs treated with DMSO, rapamycin (20 nM), Deltazinone 1 (6 µM), or Deltasonamide 1 (6 µM) for 24 h. Left, lysates were characterized by immunoblotting of the indicated proteins. Right, densitometric analysis of phospho-S6K levels normalized to S6K total levels. β-actin, loading control **(B)**. *Tsc2-null* MEFs treated with DMSO or Deltasonamide 1 (0.5–8 µM) for 24 h. Left, lysates were characterized by immunoblotting of the indicated proteins. Right, the dose-response curve for *Tsc2-null* MEFs treated with Deltasonamide 1 for 24 h. Normalized pS6K levels at each drug concentration is shown as a percentage of the normalized pS6K levels with DMSO. IC_50_, half-maximal inhibitory concentration **(C)**. *Tsc2-null* MEFs treated as in **(A)**. Lysates were characterized by immunoblotting of the indicated proteins. Phospho-4EBP1 (Thr37/46), phospho-4EBP1 (Thr70), 4EBP1 total and β-actin (loading control). Results are representative of at least three independent experiments. Data are expressed as the mean ± SD (*p* < 0.05).

**FIGURE 4:**
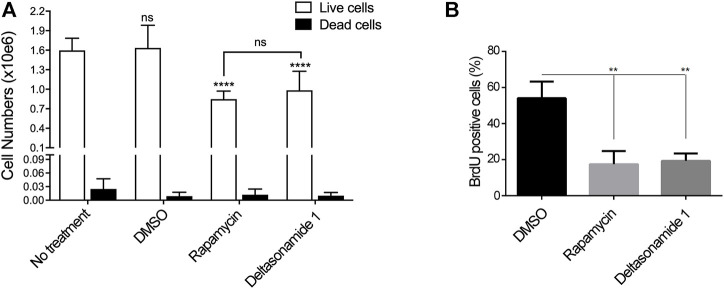
Deltasonamide 1 inhibits *Tsc2-null* MEFs proliferation **(A)**. *Tsc2-null* MEFs treated with DMSO, Deltasonamide 1 (6 µM) or rapamycin (20 nM) for 24 h. After drug treatment, live and dead cell numbers were marked with Live/DeadTM Fixable Green Dead Cell staining assay and analyzed with an automated cell counter **(B)**. *Tsc2-null* MEFs treated as in **(A)**. The graph shows the percentage of 5-Bromo-2-deoxyuridine (BrdU) positive cells after treatment. Results are representative of at least three independent experiments. Data are expressed as the mean ± SD (*p* < 0.05).

Because the binding of either Deltasonamide 1 or Deltazinone 1 to PDEδ disrupts the interaction between PDEδ and Rheb in yeast, we sought to explore whether compound treatment could affect the function of Rheb in mammalian cells. For this purpose, we analyzed the regulation of mTORC1 as the main downstream target of Rheb (Sato et al., 2008), in presence or absence of Deltasonamide 1 or Deltazinone 1. We used *Tsc2-null* MEFs where the upregulation of mTORC1 signaling depends on Rheb hyperactivation (Zhang H. et al., 2003). We initially evaluated the effect of the compounds on mTORC1 signaling by measuring the phosphorylation of the S6 protein, a downstream target of this pathway (Holz et al., 2005). Deltasonamide 1 treatment dose-dependently reduced S6 phosphorylation (pS6) in a concentration range between 0.5 and 6 µM (Figure 2A-B). Surprisingly, despite the potent inhibition of the association of Rheb and PDEδ by Deltazinone 1 in yeast, the compound did not affect S6 phosphorylation (pS6) in *Tsc2-null* MEFs at concentrations up to 6 µM (data not shown). Immunocytochemistry assays showed that the reduction of pS6 levels by Deltasonamide 1 is already evident after 4 h of incubation and persists at 24 h (Figure 2C). This result was confirmed by Western blot analysis, where rapamycin, a known mTORC1 inhibitor was used as a positive control of mTORC1-dependent signaling regulation (Figure 2D). We next analyzed the phosphorylation levels of S6K (Figures 3A,B) and 4EBP1 (Figure 3C), two direct downstream targets of mTORC1. As expected, increasing doses of Deltasonamide 1 in *Tsc2-null* MEFs resulted in a dose-dependent decrease on mTORC1-dependent S6K phosphorylation at threonine 389 with an IC_50_ of 4.49 µM (Figure 3A). Deltazinone 1 was again ineffective (Figure 3B). In contrast to Rapamycin treatment, we could not detect a significant effect of Deltasonamide 1 on 4EBP1 phosphorylation as judged by the top band signal on Western blots (Figure 3C and Supplementary Figure S3). However, changes in the electrophoretic pattern of the total and phosphorylated 4EBP1 (p4EBP1) were evident. Like Rapamycin, Deltasonamide 1 treatment caused the appearance of lower molecular weight bands on Western blots either with the total or the phosphorylation antibodies consistent with reduced phosphorylation of 4EBP1 due to mTORC1 inhibition (see discussion). Confirming previous results, the Deltazinone 1 compound was ineffective on the pS6 and p4EBP1 levels (Figure 3C). Overall, our results demonstrated that Deltasonamide 1 negatively regulates Rheb/mTORC1 signaling in *Tsc2-null* MEFs.

### 3.3 Deltasonamide 1 Reduces Cell Proliferation in *Tsc2-Null* MEFs

The Rheb/mTORC1 pathway promotes protein synthesis, cell growth, and cell proliferation through the activation of S6K and inhibition of the translational repressor protein 4EBP1 (Ben-Sahra and Manning, 2017). Since Deltasonamide 1 impaired Rheb/mTORC1 signaling in cells, we evaluated the effect of Deltasonamide 1 on cell proliferation. Deltasonamide 1 or rapamycin significantly decreased cell numbers after 24 h of treatment compared to the control group (DMSO) in *Tsc2-null* MEFs (*p* < 0.05), an effect not associated with an increase in dead cells (Figure 4A). In agreement with a reduction on cell proliferation, 24 h treatment with Deltasonamide 1, as well as rapamycin, significantly reduced 5-Bromo-2′-deoxiuridine (BrdU) incorporation in *Tsc2-null* MEFs (Figure 4B). These results demonstrated that Deltasonamide 1 has an antiproliferative effect on *Tsc2-null* MEF cells.

### 3.4 Prolonged Deltasonamide 1 Treatment Increases Cell Death in *Tsc2-Null* MEFs

**FIGURE 5:**
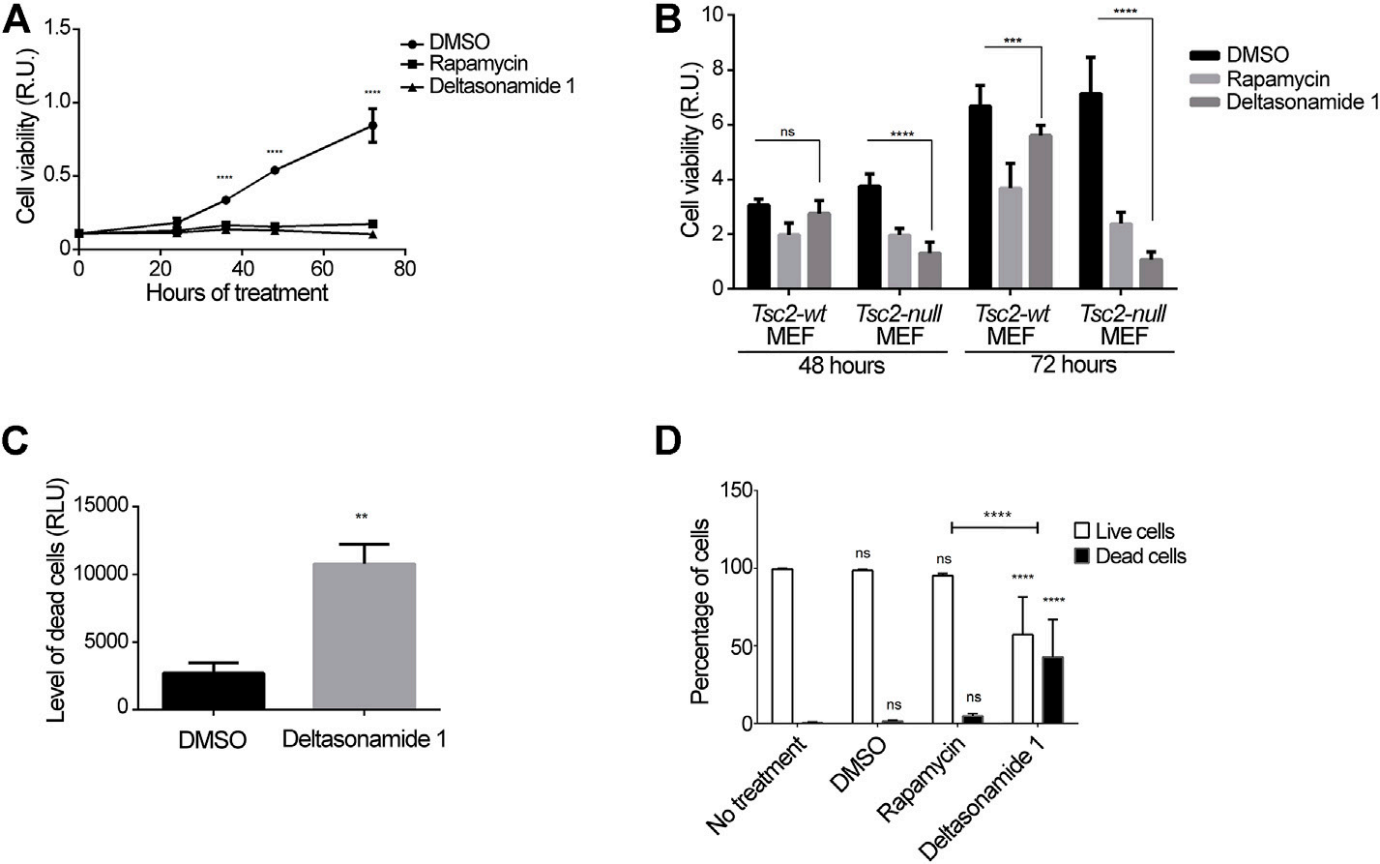
Prolonged Deltasonamide 1 treatment increases *Tsc2-null* MEFs death **(A)**. *Tsc2-null* MEFs treated with DMSO, rapamycin (20 nM), or Deltasonamide 1 (6 µM) for 24 h up to 72 h. After treatment, cell viability was measured by XTT assay **(B)**. *Tsc2-wt* and *Tsc2-null* MEFs treated with DMSO, rapamycin (20 nM) or Deltasonamide 1 (6 µM) for 48 and 72 h. After treatment, cell viability was measured by XTT assay **(C)**. *Tsc2-null* MEFs treated with DMSO or Deltasonamide 1 (6 µM) for 48 h. After treatment, number of dead cells was estimated using the CytoTox-GloTM kit Live/DeadTM assay **(D)**. *Tsc2-null* MEFs treated with DMSO, rapamycin (20 nM), or Deltasonamide 1 (6 µM) for 48 h. Live and dead cells with each drug treatment were marked with Live/DeadTM Fixable Green Dead Cell staining assay and analyzed with an automated cell counter. Results are representative of at least three independent experiments. Data are expressed as the mean ± SD (*p* < 0.05).

Finally, we evaluated the cytotoxic effect of prolonged Deltasonamide 1 treatment. First, we used XTT assay to measure viable cells after incubation with Deltasonamide 1, rapamycin, or vehicle (DMSO) up to 72 h. After 24 h, we found that Deltasonamide 1 and rapamycin significantly diminished the *Tsc2-null* MEFs viability (Figure 5A). We also tested whether the drug could affect the viability of *Tsc2 wild type* cells (*Tsc2-wt* MEFs). Interestingly, only rapamycin significantly affected the *Tsc2-wt* MEFs viability at 48 h incubation (Figure 5B). Deltasonamide 1 reduced *Tsc2-wt* MEFs viability at 72 h, but this effect was less robust than in the *Tsc2-null* MEFs. These results suggest that Rheb/mTORC1-dependent cells such as *Tsc2-null* MEFs, are more susceptible to the anti-viability effects of Deltasonamide 1.

These results suggest that mTORC1 is more dependent on Rheb activity in *Tsc2-null* MEFs. Consistently, rapamycin, but not Deltasonamide 1, significantly affected mTORC1 activity in *Tsc2*-*wt* MEFs (Supplementary Figure S4). Because the XTT assay evaluates cell viability according to cell metabolic state and membrane integrity, we could not rule out that the observed effects are due to a reduction on cell metabolism by the inhibition of the mTORC1 activity. Thus, we also evaluated cell death using an assay only based on cell membrane integrity (CytoTox-Glo™ kit and Live/Dead™ Fixable Green Dead Cell staining assay). Consistent with the cell viability findings in Figure 5, Deltasonamide 1 significantly increased *Tsc2-null* MEF dead cells after 48 h of treatment (Figure 5C-D). By contrast, rapamycin did not significantly affect the percentage of dead cells under similar conditions (Figure 5D). Altogether, our results suggest that Deltasonamide 1-mediated inhibition of Rheb rather than mTORC1 specifically affects the survival of *Tsc2-null* MEFs.

## 4 Discussion

Rheb is a small GTPase involved in cell growth by stimulating mTORC1 activity (Long et al., 2005a; Sato et al., 2008). The Rheb/mTORC1 signaling pathway is upregulated in the TSC syndrome and several human cancers (Carsillo et al., 2000; Lassman et al., 2005; Crino et al., 2006; Nardella et al., 2008; Lu et al., 2010). Hence, targeting Rheb is a rational strategy to develop therapies against proliferative cell disorders where Rheb and, consequently, mTORC1 activity are upregulated. Recently, Mahoney et al. showed the NR1 compound, a Rheb inhibitor that blocks Rheb-mTORC1 interaction but does not affect mTORC2 after chronic treatment (Mahoney et al., 2018), like other mTOR inhibitors (Sarbassov et al., 2006). Although NR1 is a promising specific Rheb inhibitor, its clinical pharmacological behavior and potential side effects are still not evaluated (Mahoney et al., 2018).

Another therapeutic strategy to target small Ras proteins, like Rheb, it is the use of PDEδ inhibitors (Spiegel et al., 2014; Schmick et al., 2015). Here, we introduced the Deltasonamide 1 agent, a new PDEδ inhibitor (Martín-Gago et al., 2017), as an alternative to target the Rheb/mTORC1 hyperactivated pathway. Our results indicated that Deltasonamide 1 affects Rheb/PDEδ interaction and is biologically stable and penetrates cellular membranes in mammalian cells. PDEδ is a cytosolic solubilization factor that transports prenylated Ras proteins from the cytoplasm back to the endomembranous system, where PDEδ-cargo association is interrupted by the small GTPase Arl2 or Arl3 (Hanzal-Bayer et al., 2002; Ismail et al., 2011; Schmick et al., 2015). Small molecules designed to impair the PDE6δ system would negatively impact signaling pathways regulated by PDEδ-farnesylated cargos (Hanzal-Bayer et al., 2002). The published PDEδ inhibitors, Deltarasin, Deltazinone 1, and the Deltasonamide 1 and 2, are hydrophobic molecules that interact with the farnesyl-binding pocket of PDEδ and are designed to target KRas oncogene-dependent signaling (Zimmermann et al., 2013; Papke et al., 2016; Klein et al., 2019). Interestingly, Rheb appeared to be more sensitive than KRas to PDEδ inhibition (Papke et al., 2016). Unlike other Ras members containing hypervariable regions, Rheb does not possess additional membrane anchorage signals, resulting in its weak association with endomembranes (Schmick et al., 2015; Papke et al., 2016). This weak association leads Rheb to constantly dissociate from membranes and, consequently, be more dependent on the PDEδ/Arl2 system than other PDEδ-farnesylated cargos (Chandra et al., 2011; Schmick et al., 2015; Papke et al., 2016). Accordingly, PDEδ silencing has more effect on the intracellular Rheb distribution than other Ras proteins (Chandra et al., 2011; Papke et al., 2016). Despite this, using si/shRNA to downregulate PDEδ as an approach to support data obtained with Deltasonamide 1 would not be informative about the specificity of this drug inhibitor. PDEδ is a highly promiscuous protein that recognizes several prenylated cargos in cells (Zhang et al., 2004 , 2005; Chandra et al., 2011; Ismail et al., 2011). Instead, Deltasonamide 1 preferentially obstructed the formation of PDEδ complexes with Rheb or KRas rather than PDEδ association with INPP5E, GRK1, Arl2, or Arl3. In addition, because of the nonspecific effect of the PDEδ silencing, we should expect that it would be cytotoxic, independently of the Rheb/mTOR-dependent cell context. On the contrary, we showed that Deltasinomide 1 caused specific cell death in the Rheb-dependent *Tsc2-null* MEFs, suggesting a more selective effect. Therefore, although we did not evaluate PDEδ silencing, it is expected that Deltasonamide 1, more specifically and efficiently, affects deregulated Rheb signaling. In agreement with an effect on the Rheb signaling by inhibiting its interaction with the PDEδ/Arl2/3 system, Deltasonamide 1 disrupted the intracellular Rheb distribution in mammalian cells (Supplementary Figure S5).

In *Tsc2-null* MEFs, Deltasonamide 1 treatment reduced phosphorylation levels of mTORC1 targets, S6 and S6K. This drug effect is likely caused by altering Rheb signaling because we previously proved that mTORC1 activation is dependent on Rheb in these cells (Lacher et al., 2010). However, in different genetic cell contexts, we cannot discard that inhibition of the KRas function could contribute to the effect of Deltasonamide 1 on the mTORC1 signaling (Byun et al., 2019).

Compared to the Deltasonamide 1 concentration (0.397 µM) that blocked Rheb-PDEδ interaction in yeast, higher concentrations were required in *Tsc2-null* MEF cells (IC_50_ = 4.49 µM) to inhibit Rheb-dependent mTORC1 signaling. Similar responses were reported for Deltarasin, an effect attributed to active function and efflux by ABC transporter proteins although we cannot exclude other mechanisms in *Tsc2-null* MEF cells (Zimmermann et al., 2013). At least for cancer cell lines, it is known that PDEδ is highly expressed up to single digit micromolar ranges (Yelland et al., 2022). Hence, equally high concentrations of PDEδ inhibitors are needed to interfere with the interaction of PDEδ and its client proteins.

In the case of Deltazinone 1, we could not detect any effect of the compound on mTORC1 signaling. This inefficacy of Deltazinone 1 is unlikely due to inefficient cell permeation or enhanced drug metabolization (data not shown) as the compound has shown profound effects on Ras-dependent functions in a variety of cell types (Papke et al., 2016). However, it may be explained by fast inhibitor release by Arl2 (Martín-Gago et al., 2017), probably requiring much higher concentrations than Deltasonamide 1 to affect Rheb localization and the mTORC1 signaling in the *Tsc2-null* MEFs. In agreement with this possibility, Deltazinone 1 required a much higher concentration (20 µM) to modestly inhibit EGF-induced S6 phosphorylation in a human pancreatic adenocarcinoma cell line and was ineffective in another pancreatic cancer cell line tested (Papke et al., 2016).

Deltasonamide 1 treatment in *Tsc2-null* MEFs changed the Western blotting pattern of total and phosphorylated 4EBP1. Other investigators reported similar observations in cells treated with rapamycin (Thoreen et al., 2009; Ducker et al., 2014; Qin et al., 2016). This effect was associated, in part, with reduced phosphorylation of 4EBP1 possibly related to a reduction of phospho-Ser65 signal, but not phospho-Thr37/46 or phospho-Thr70 (Thoreen et al., 2009; Ducker et al., 2014; Qin et al., 2016). At least seven phosphorylation sites are reported for 4EBP1; four phosphorylated by mTORC1 (Thr36, Thr47, Ser65, and Thr70) (Qin et al., 2016). However, unlike S6K, the inhibition of mTORC1 by rapamycin or its analogs only partially affects 4EBP1 and does not affect all residues phosphorylated by mTORC1 (Thoreen et al., 2009; Ducker et al., 2014; Qin et al., 2016). In this work, Ser65 phosphorylation of 4EBP1 was not evaluated, but phosphorylation of Thr37, Thr46, and Thr70, as well as total 4EBP1, showed additional bands of lower molecular weight after Deltasonamide 1 treatments, which is likely associated with reduced phosphorylation of other 4EBP1 residues. Similar effects on the banding pattern of 4EBP1 were reported for treatment with the Rheb inhibitor NR1, which the authors related to inhibition of Rheb and mTORC1 (Mahoney et al., 2018).

Rheb is involved in the proliferation and survival of several cancer cell lines (Campos et al., 2016; Tian et al., 2020). Accordingly, we observed that Deltasonamide 1 treatment decreased the proliferation and survival of *Tsc2-null* MEF cells. An effect of the drug on proliferation due to inhibition of Rheb function is consistent with our previous studies showing that Rheb depletion inhibited the proliferation of *Tsc2-null* cells (Lacher et al., 2010). Interestingly, this inhibition was associated with a mTORC1-independent increase of p27 levels and reduction of Cdk2 activity, and with inhibition of tumorigenesis (Lacher et al., 2010).

Although Deltasonamide 1 has obvious limitations in cell permeability, it appears to be an excellent *in vitro* tool compound, which may be moved into *in vivo* DMPK (Drug Metabolism and Pharmacokinetic) tolerability and safety experiments, potentially followed by efficacy testing in TSC animal models and on tumor growth in mice. Our observation that prolonged incubation with Deltasonamide 1 did not impair *Tsc2-wt* MEFs viability to the same extent as the *Tsc2-null* MEFs suggests pronounced cytotoxic effect on cells with Rheb-dependent upregulation of mTORC1. This conclusion is supported by the modest effect of Deltasonamide 1 on the Rheb/mTORC1 axis in *Tsc2-wt* MEFs. The cytotoxic effect of Deltasonamide 1, unlike rapamycin, in *Tsc2-null* MEF cells suggests mTORC1-independent functions of Rheb affecting cell survival (Armijo et al., 2016), such as the regulation of AMPK (Lacher et al., 2010; Campos et al., 2016), Notch signaling (Karbowniczek et al., 2010), and activation of Carbamoyl-phosphate synthetase 2, Aspartate transcarbamoylase, and Dihydroorotase (CAD) protein (Sato et al., 2015). Nevertheless, the full involvement of PDEδ inhibition by Deltasonamide 1 in signal transduction pathways remains to be fully elucidated. Additional studies will help shed light on the therapeutic potential of Deltasonamide 1 to proliferative disorders associated with upregulation of Rheb/mTORC1 signaling.

## Data Availability

The original contributions presented in the study are included in the article/Supplementary Material, further inquiries can be directed to the corresponding authors.
