# Supplementary material for: Blocking the Farnesyl Pocket of PDEδ Reduces Rheb-Dependent mTORC1 Activation and Survival of Tsc2-Null Cells
Source: Front Pharmacol. 2022 Jun 23;13:912688. doi: 10.3389/fphar.2022.912688 (PMC9260180; doi:10.3389/fphar.2022.912688)
Supplement: Supplementary file 1 [file DataSheet1.pdf]

Supplementary Material

1. Supplementary Figures

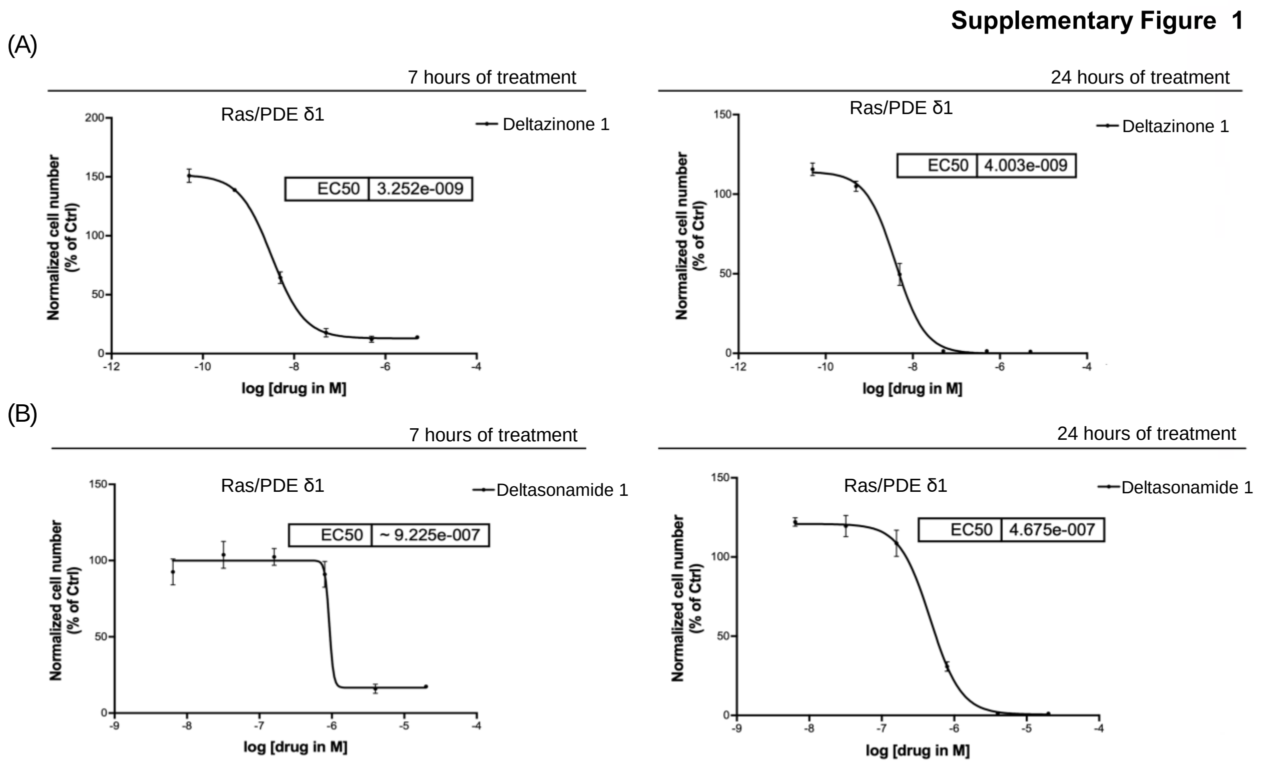

Supplementary Figure 1. (**A**). Yeast two hybrid LacZ quantitative measurements for the interactions of Ras/PDEδ. Diploid yeast cells containing bait and prey constructs for Ras/PDEδ were grown on selective media lacking tryptophan and leucine in the presence of the β-galactosidase substrate ONPG and Deltazinone 1 at titrated compound concentrations (5 μM highest concentration) for 7 h (upper panel) or 24 h (lower panel) incubation prior to the measurement of β-galactosidase activity. (**B**). As in (A) for Deltasonamide 1 (20 μM highest concentration). IC_{50}, half-maximal effective concentration. Results are representative of at least three independent experiments. Data are expressed as the mean ± SD. ($p < 0.05$).

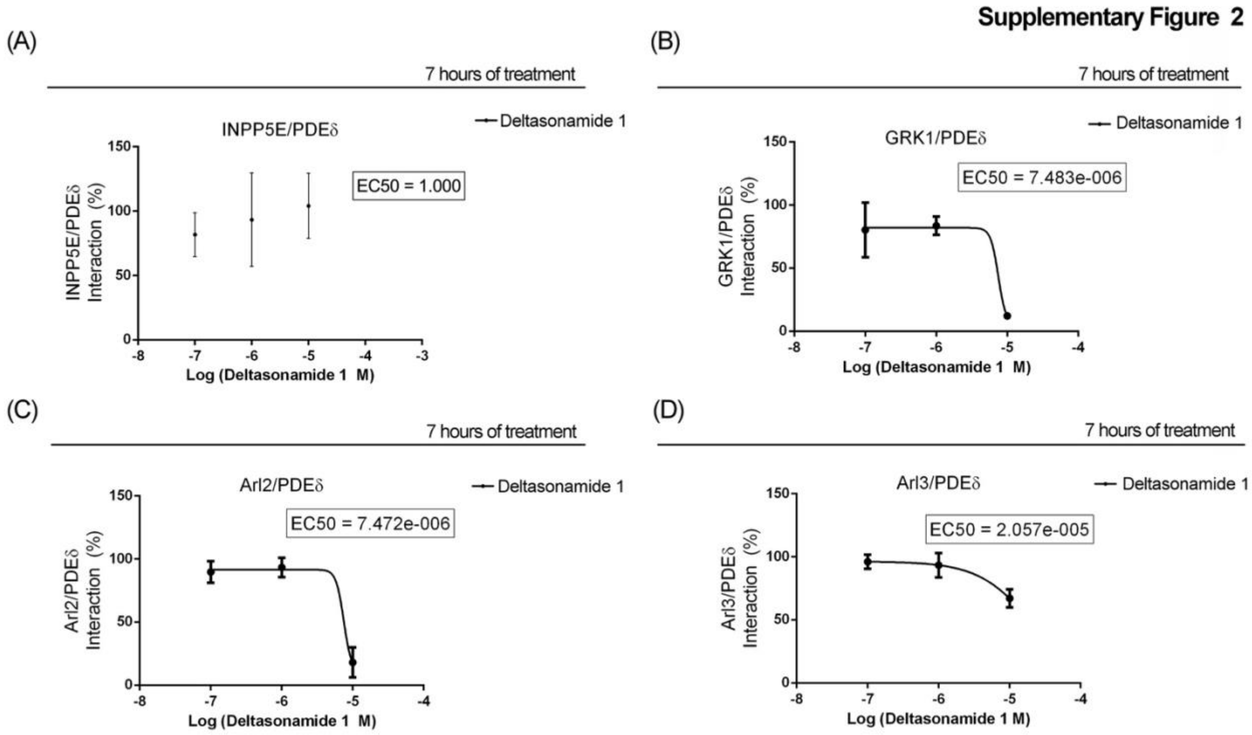

Supplementary Figure 2. Yeast two hybrid LacZ quantitative measurements for the interactions of PDEδ with different proteins. (**A**) Diploid yeast cells containing bait and prey constructs for INPP5E/PDEδ were grown on selective media lacking tryptophan and leucine in the presence of the β-galactosidase substrate ONPG and Deltasonamide 1 at titrated compound concentrations (0.1, 1.0 and 10 μM concentration) for 7 h incubation prior to the measurement of β-galactosidase activity. (**B**). As in (A) for cells containing bait and prey constructs for GRK1/PDEδ. (**C**). As in (A) for cells containing bait and prey constructs for Arl2/PDEδ. (**D**). As in (A) for cells containing bait and prey constructs for Arl3/PDEδ. IC_{50}, half-maximal effective concentration. Results are representative of at least three independent experiments. Data are expressed as the mean ± SD. ($p < 0.05$).

Supplementary Figure 3

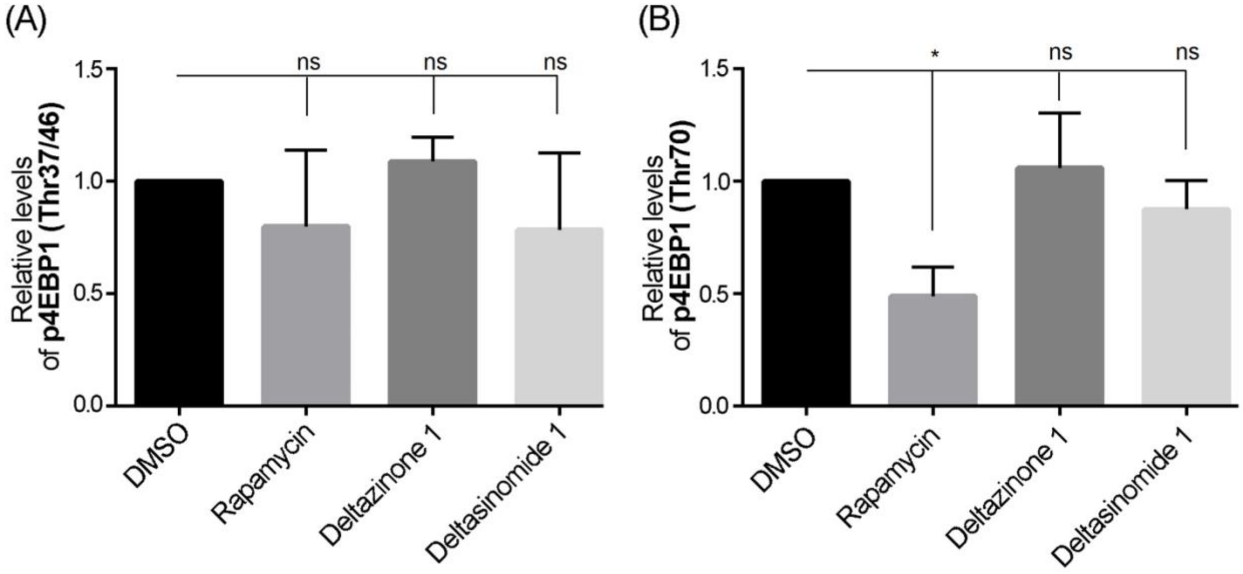

Supplementary Figure 3. Quantifications of Western blot results from figure 3C. (**A**) Densitometric analysis of phospho-4EBP1 (Thr37/46) levels normalized to 4EBP1 total levels. β-Actin, loading control. (**B**) Same as (A), but for phospho-4EBP1 (Thr70). Results are representative of at least three independent experiments. Data are expressed as the mean ± SD. ($p < 0,05$).

Supplementary Figure 4

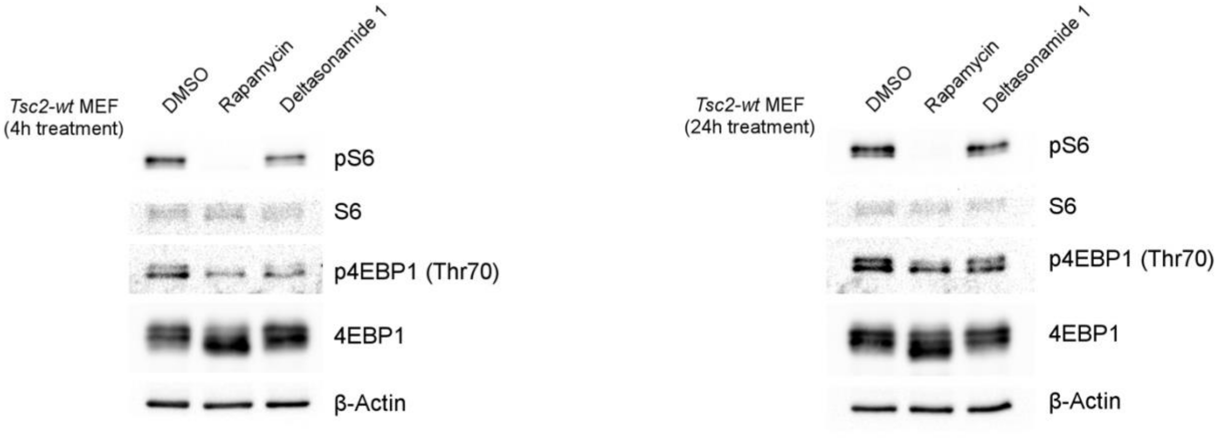

Supplementary Figure 4. Deltasonamide 1 did not affect the mTORC1 signaling in *Tsc2-wt* MEFs. *Tsc2-wt* MEFs treated with DMSO, rapamycin (20 nM), or Deltasonamide 1 (6 µM) for 4 h (*left panel*) and 24 h (*right panel*). Lysates were characterized by immunoblotting of the indicated proteins. Results are representative of three independent experiments.

Supplementary Figure 5

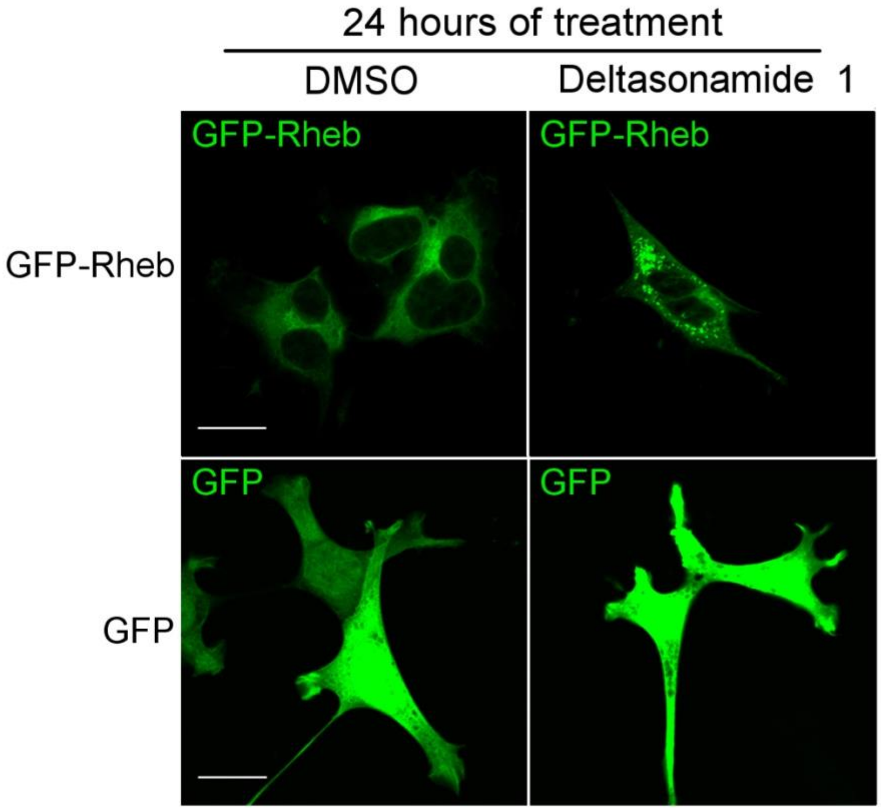

Supplementary Figure 5. Deltasonamide 1 affects the cellular localization of Rheb. *Tsc2-wt* MEF cells were transfected with pEGFP-C1-GFP-RhebWT (GFP-Rheb) or pEGFP-C1 (GFP only) and treated with 6µM Deltasonamide 1 or DMSO for 24 hours. GFP-Rheb and GFP levels were analyzed by immunofluorescence and confocal microscopy. Scale bar = 20 µm.
